# Clearance of mixed biofilms of *Streptococcus pneumoniae* and methicillin-susceptible/resistant *Staphylococcus aureus* by antioxidants *N*-acetyl-l-cysteine and cysteamine

**DOI:** 10.1038/s41598-022-10609-x

**Published:** 2022-04-23

**Authors:** Julio Sempere, Mirella Llamosí, Federico Román, Darío Lago, Fernando González-Camacho, Covadonga Pérez-García, Jose Yuste, Mirian Domenech

**Affiliations:** 1 Spanish Pneumococcal Reference Laboratory, National Centre for Microbiology, Instituto de Salud Carlos III, Madrid, Spain; 2 CIBER de Enfermedades Respiratorias (CIBERES), Madrid, Spain; 3 Intrahospital Infections Unit, National Centre for Microbiology, Instituto de Salud Carlos III, Madrid, Spain; 4 Department of Genetics, Physiology, and Microbiology, Faculty of Biology, Complutense University of Madrid, Madrid, Spain

**Keywords:** Biological techniques, Drug discovery, Microbiology, Diseases, Pathogenesis

## Abstract

Biofilm-associated infections are of great concern because they are associated with antibiotic resistance and immune evasion. Co-colonization by *Staphylococcus aureus* and *Streptococcus pneumoniae* is possible and a threat in clinical practice. We investigated the interaction between *S. aureus* and *S. pneumoniae* in mixed biofilms and tested new antibiofilm therapies with antioxidants *N*-acetyl-l-cysteine (NAC) and cysteamine (Cys). We developed two in vitro* S. aureus*–*S. pneumoniae* mixed biofilms in 96-well polystyrene microtiter plates and we treated in vitro biofilms with Cys and NAC analyzing their effect by CV staining and viable plate counting. *S. pneumoniae* needed a higher proportion of cells in the inoculum and planktonic culture to reach a similar population rate in the mixed biofilm. We demonstrated the effect of Cys in preventing *S. aureus* biofilms and *S. aureus*–*S. pneumoniae* mixed biofilms. Moreover, administration of 5 mg/ml of NAC nearly eradicated the *S. pneumoniae* population and killed nearly 94% of MSSA cells and 99% of MRSA cells in the mixed biofilms. The methicillin resistance background did not change the antioxidants effect in *S. aureus*. These results identify NAC and Cys as promising repurposed drug candidates for the prevention and treatment of mixed biofilms by *S. pneumoniae and S. aureus*.

## Introduction

*Streptococcus pneumoniae* colonizes the nasopharyngeal tract asymptomatically of 5–10% of adults and 20–40% of children^1,2^. The burden of disease by *S. pneumoniae* is substantial as it is the main bacterial cause of community-acquired pneumonia, acute otitis media (AOM), bacterial meningitis, and a major cause of bacterial sepsis^3,4^. Antibiotics are essential players against pneumococcal infections, but the emergence of multidrug-resistant strains^5–7^ makes preventive measures such as vaccines one of the best cost-effective strategies to ameliorate the impact of antibiotic resistance in the epidemiology of *S. pneumoniae*^6^. Pneumococcal vaccines have decreased the incidence of invasive pneumococcal disease (IPD) worldwide, but the emergence of non-vaccine serotypes, due to serotype replacement and capsular switch phenomena, is worrisome in most of the European countries^8–11^. In addition, the impact of pneumococcal vaccines against colonization of the nasopharynx seems to be moderate in the pediatric population^12^.

Nasopharyngeal colonization, a critical step in IPD, is associated with biofilm formation, where the capsular polysaccharide (CPS) of *S. pneumoniae*, can be an impediment. The expression of CPS is detrimental for biofilm formation^13,14^. At least 101 different pneumococcal serotypes are known demonstrating the high variability of CPS within this pathogen^15,16^. The serotype 19A, which is a good biofilm former in vitro, is also a good colonizer with a significant incidence of IPD^9–11,17,18^. The important rise in the last years of antibiotic-resistant strains of serotype 19A since the introduction of the PCVs^19–21^ makes this particular serotype an ideal candidate for biofilm studies.

*Staphylococcus aureus* frequently colonizes the skin of the human population (> 30%) but can be found in the nasopharynx up to 80% of individuals being serotypes 5 and 8 among the most frequent producing staphylococcal infections^22–25^. *S. aureus* is well known for producing implant-associated infections, where the biofilm state is essential for promoting persistence, evasion of the immune system, and antimicrobial resistance^26^. Furthermore, this pathogen can produce secondary episodes of bacterial pneumonia after influenza virus infection^22,27,28^, which is a frequent trait shared with *S. pneumoniae*^29^. In addition, the emergence of methicillin-resistant *Staphylococcus aureus* (MRSA) is worrisome in the hospital environment because it is associated with high mortality rates^30^. This situation can get worse when the infection is linked to biofilms due to the increased resistance and competence, leading to the dissemination of antibiotic resistance genes between MRSA and methicillin-susceptible *Staphylococcus aureus* (MSSA) strains and even with non-related microorganisms in a mixed biofilm^26^. In 2004, Cryer et al*.* when studying patients with recalcitrant chronic rhinosinusitis demonstrated the existence of biofilms on the sinus mucosa^31^. In addition, mixed infections between *S. aureus* and other pathogens such as *S. pneumoniae* producing biofilm-associated pathologies including otitis, rhinitis, and sinusitis have been reported^32–35^.

During the pre-vaccine era and after the introduction of PCV7, several studies have shown a negative association between *S. aureus* and *S. pneumoniae* carriage, describing that carriage of PCV7 vaccine serotypes impairs the colonization by *S. aureus*^2,36–38^. The mechanism described for this negative association was observed using in vitro studies and was related to the killing of *S. aureus* with hydroxyl radicals (·OH) released by the presence of pneumococcus, which induces DNA degradation, leading to the death of *S. aureus* strains^39^. In contrast, co-colonization in vitro and in vivo studies have demonstrated that this negative association does not occur in a mixed biofilm over a cell layer^4^ and in animal models^40,41^. In this sense, epidemiological studies showed that up to 24% of patients were colonized by both species^42–44^ and that vaccination with the conjugate vaccine did not modify the nasopharyngeal carriage by *S. aureus*^45,46^.

Due to discrepancies in the literature described above, the interaction between *S. pneumoniae* and *S. aureus* in biofilm-related diseases needs to be investigated in more detail. The physiological interactions between bacteria involved in multispecies biofilms include the exchange of genetic material and antibiotic resistance factors^47^. Recalcitrance is a common characteristic of a biofilm in which the concentration of antibiotics necessary to clear the bacteria can be up to 1,000 times higher in the biofilm than against planktonic microorganisms^48^. Moreover, there are insufficient data supporting potential therapies that can target both microorganisms causing infection in a biofilm state. In this sense, several evidences suggest that antioxidants compounds such as *N*-acetyl-l-cysteine (NAC) and cysteamine (Cys) might have the potential as antimicrobial drugs against individual biofilms of certain species, although their activity against polymicrobial biofilms of *S. aureus* and *S. pneumoniae* is unknown^49–54^.

In this work, we describe two in vitro models of an MSSA-*S. pneumoniae *(*Sp*) and MRSA-*Sp* mixed biofilms that can be useful to understand the dynamics of the interaction between these two pathogens and to test antibiofilm therapies. Hence, the antimicrobial activity of NAC and Cys has been explored in both mixed biofilm models.

## Results

### Mixed biofilms of *S. pneumoniae* and *S. aureus* require a higher proportion of pneumococcal cells and a short incubation period

**Figure 1:**
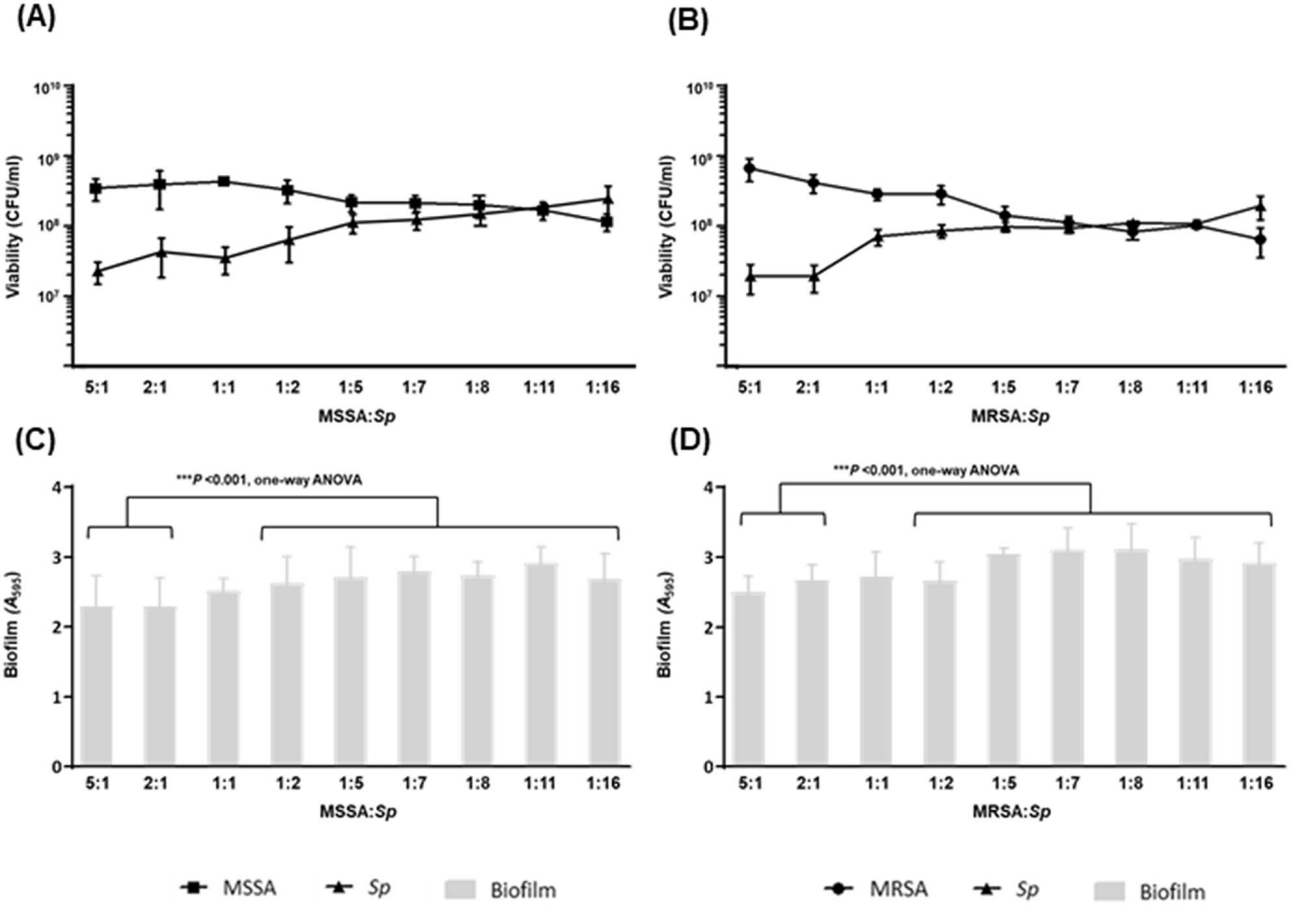
Influence of bacterial proportions in the inoculum on MSSA/MRSA-*Sp* mixed biofilm formation in vitro. *S. pneumoniae* and *S. aureus* were mixed in different proportions, and aliquots of 200 µl final volume containing from 4 × 10^5^ to 4 × 10^6^ CFU/ml were dispensed into 96-well polystyrene microtiter plates and incubated 5 h at 34 °C. (**A**,**B**) The viability of MSSA (squares), MRSA (circles) and *S. pneumoniae* (triangles) was determined by plate counting. (**C**,**D**) Grey bars indicate mixed biofilm formation determined by CV staining (biomass). Data represent the average of at least three experiments. Standard deviation bars are shown.

We first examined the influence of different proportions of pneumococcal and MSSA/MRSA bacterial cells in the inoculum to establish the optimal conditions for the survival of both species in the mixed biofilm (Fig. 1). We measured the biomass using a CV assay measuring the optical density (*A*_595_) and viable count cells determined as CFU/ml in mixed biofilms (Fig. 1). To obtain a similar level of both species (*S. pneumoniae* and *S. aureus*) in the mixed biofilm at the final time, the proportion of *S. pneumoniae* in the inoculum had to be much higher than *S. aureus* regardless of the susceptibility profile (MSSA or MRSA) (Fig. 1A,B). Moreover, we found a significantly increased pattern in the biofilm biomass (*A*_595_) when the proportion of *S. pneumoniae* in the inoculum was higher (proportions 1:2, 1:5, 1:7, 1:8, 1:11, and 1:16) in comparison to greater levels of *S. aureus* in the inoculum (proportions 5:1 and 2:1) in both systems (***P < 0.001, one-way ANOVA) (Fig. 1C,D).

**Figure 2:**
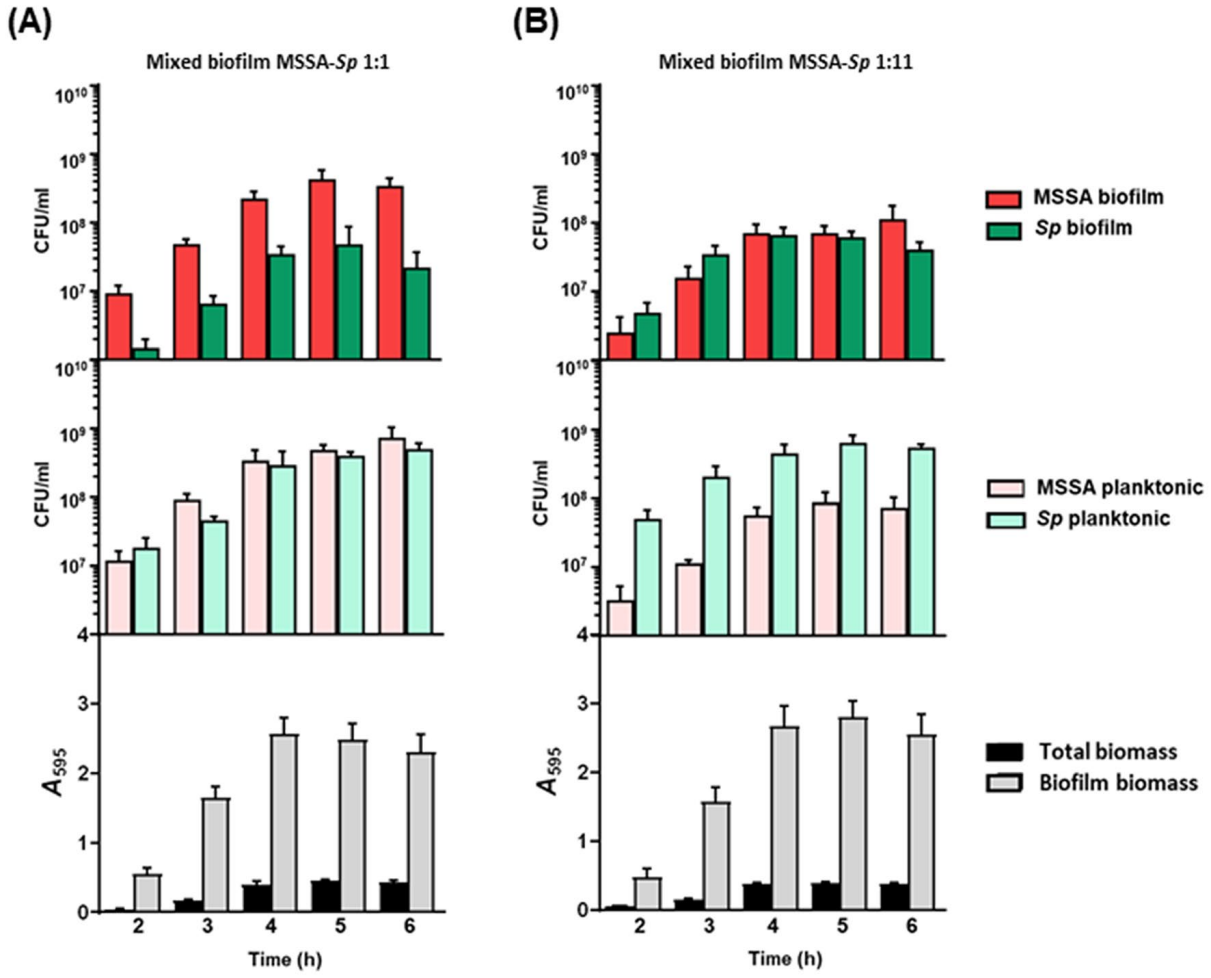
Time course of MSSA-*Sp* mixed biofilm formation using two proportions. (**A**) Mixed biofilm MSSA-*Sp* 1:1. (**B**) Mixed biofilm MSSA-*Sp* 1:11. In the top panel is represented MSSA (red) and *S. pneumoniae* (green) viable cells within the biofilm. In the middle panel is represented MSSA viable cells (light red) and *S. pneumoniae* (light green) viable cells in the planktonic culture. In the bottom panel is represented the total biomass (black bars) and biofilm biomass (grey bars) determined by CV staining. The data represent the average of six experiments. Standard deviation bars are shown.

**Figure 3:**
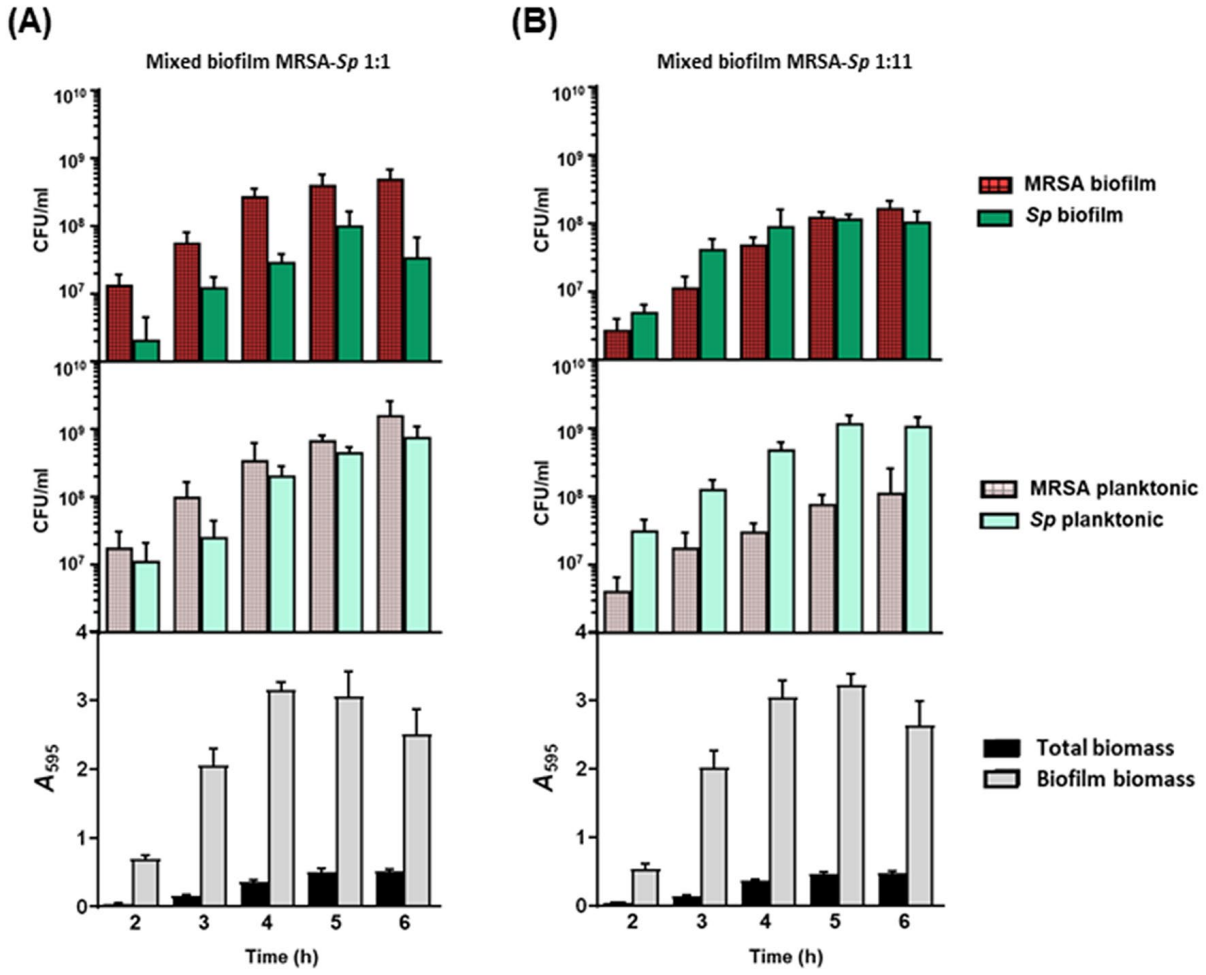
Time course of MRSA-*Sp* mixed biofilm formation using two proportions. (**A**) Mixed biofilm MRSA-*Sp* 1:1. (**B**) Mixed biofilm MRSA-*Sp* 1:11. In the top panel is represented MRSA (patterned red) and *S. pneumoniae* (green) viable cells within the biofilm. In the middle panel is represented MSSA viable cells (light patterned red) and *S. pneumoniae* (light green) viable cells in the planktonic culture. In the bottom panel is represented the total biomass (black bars) and biofilm biomass (grey bars) determined by CV staining. The data represent the average of six experiments. Standard deviation bars are shown.

For further experiments, we chose the proportions 1:1 and 1:11 for all the mixed biofilms (MSSA:*Sp* and MRSA:*Sp*) and the incubation period of 5 h because individual biofilms of both species showed a reduction in the viability after 5–6 h (Fig. S1). The choice of the 1:1 proportion was useful to test NAC and Cys in an inhibition assay because, at the early phase of biofilm formation, both populations in the inoculum are equal, an ideal condition to test antioxidants as a preventive measure. On the other hand, the proportion 1:11 was selected to test the therapeutic effect in a preformed mature mixed biofilm where both populations are equal at 4–5 h (Figs. 1, 2, 3).

Once the experimental conditions were established, we found that the MSSA-*Sp* and MRSA-*Sp* mixed biofilms grew at similar rates in comparison to individual biofilms (Figs. 2, 3, and Fig. S1). At the 1:1 proportion, MSSA and MRSA showed a higher ability to adhere to the polystyrene plates and form biofilms in comparison to *S. pneumoniae* 19A, whereas in planktonic cultures, the proportions of both species were similar (Figs. 2A and 3A). Our results confirm that *S. pneumoniae* needs an initial advantage, with a higher proportion of the inoculum size, to achieve a similar population level in the mixed biofilm after 4 h (Figs. 2B and 3B). In addition, a higher proportion of *S. pneumoniae* was necessary for the planktonic culture to maintain a similar population rate of both bacterial species in the mixed biofilm (Figs. 2B and 3B), whereas *S. aureus* can maintain a higher population in the mixed biofilm with a similar proportion in the planktonic culture (Figs. 2A and 3A).

### Effects of cysteamine in *S. aureus* monospecific biofilms

The antimicrobial activity of Cys in the prevention and treatment of *S. aureus* biofilms was analyzed because it has never been reported for this pathogen (Figs. S2 and S3). We used strains of both serotypes 5 and 8. In the inhibition assays (prevention), we observed a significant antimicrobial effect when Cys was used with doses over the MIC showing a dramatic reduction in the total biomass and biofilm biomass of both MSSA and MRSA strains from both serotypes (Fig. S2). In the case of the MRSA strain of serotype 5, exposure to concentrations around the MIC (0.1 mg/ml of Cys) had a stronger effect (***P < 0.001) both in the total and biofilm biomass (Fig. S2B) in comparison to the MSSA strain and both strains of serotype 8, where we only could observe a reduction in the total biomass but not in the biofilm formation (Fig. S2).

Treatment of *S. aureus* biofilms with Cys showed antimicrobial activity with concentrations ≥ 2.5 mg/ml (***P < 0.001, one-way ANOVA) with a greater effect in the strains of serotype 8 (Fig. S3). Lower concentrations were tested but did not show any effect in the preformed biofilm (data not shown). In the case of strains of serotype 5, this reduction after Cys treatment showed a plateau with increasing concentrations of Cys (Fig. S3A,B), and similar reduction levels were obtained even with higher doses such as 20 mg/ml (data not shown). However, in strains of serotype 8, higher doses such as 5 and 10 mg/ml of Cys reduced, even more, the viability of the population within the biofilm (Fig. S3C,D). In both serotypes, Cys effect was stronger in the MRSA strain than in the MSSA strain (Fig. 3). Overall, treatment with Cys was effective against the biofilm of *S. aureus* reaching from a 45% reduction in the MSSA strain of serotype 5 (Fig. S3A) to a 93% reduction in the MRSA strain of serotype 8 (Fig. S3B).

### Antibiofilm effects of NAC and Cys in MSSA-*Sp* and MRSA-*Sp* mixed biofilms

**Figure 4:**
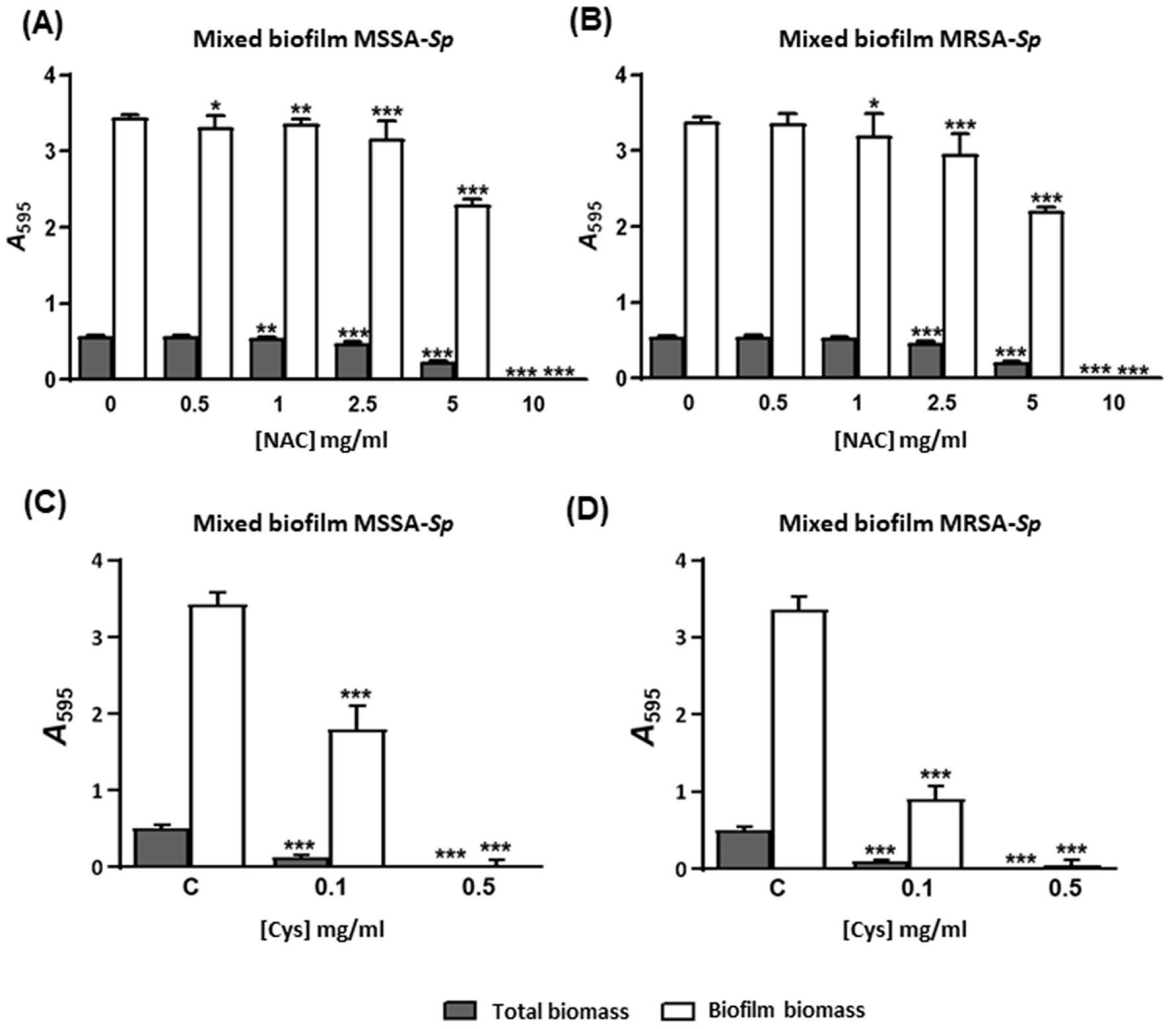
Prevention with antioxidants of MSSA/MRSA-*Sp* mixed biofilms (Inhibition of mixed biofilms). Mixed biofilms were distributed in the wells of a microtiter plate, which was then incubated for 5 h at 34 °C in the presence of different concentrations of NAC (**A**,**B**) or Cys (**C**,**D**). The MIC of NAC for the *S. pneumoniae* strain was 2.5 mg/ml and 5 mg/ml for both MSSA /MRSA strains whereas the MIC of Cys for the *S. pneumoniae* strain and both *S. aureus* strains was 0.156 mg/ml. Absorbance levels after CV staining of mixed biofilms (**A**) and (**C**) MSSA-*Sp* (**B**) and (**D**) MRSA-*Sp*. Dark grey bars represent total biomass (adherent plus non-adherent cells) and white bars represent mixed biofilm biomass. Data represent the average of at least three experiments. Standard deviation bars are shown, and asterisks mark statistically significant results (two-tailed Student’s *t* test: *P < 0.05; **P < 0.01; ***P < 0.001) when comparing the treatment versus the non-treated biofilm. For multiple comparisons, we performed one-way ANOVA obtaining ***P < 0.001 in all the cases.

We evaluated the antibiofilm activity of NAC and Cys in the prevention of mixed biofilms by *S. aureus* and *S. pneumoniae* grown in the 1:1 proportion (Fig. 4). The preventive effect of NAC in both models was lower than the effect of Cys. The use of sub-inhibitory concentrations of NAC in the mixed biofilm reduced the total biomass (adhered and not adhered cells) and biofilm biomass of both systems (Fig. 4A,B), but only after 2.5 mg/ml of NAC this effect was greater, achieving the biofilm biomass at 10 mg/ml of NAC in both systems (***P < 0.001, one-way ANOVA) (Fig. 4A,B). The use of concentrations of Cys around the MIC in the mixed biofilm reduced the total biomass and biofilm biomass, being higher in the MRSA-*Sp* mixed biofilm at 0.1 mg/ml (***P < 0.001) (Fig. 4D) than in the MSSA-*Sp* mixed biofilm (Fig. 4C). These results confirm previous findings with individual biofilms of *S. aureus* and Cys (Fig. S2A,B). Moreover, when using concentrations of Cys over the MIC, the biofilm biomass was nearly eradicated (***P < 0.001, one-way ANOVA) (Fig. 4C,D). These results, confirm Cys as an ideal candidate to prevent *S. aureus*–*S. pneumoniae* mixed biofilms.

**Figure 5:**
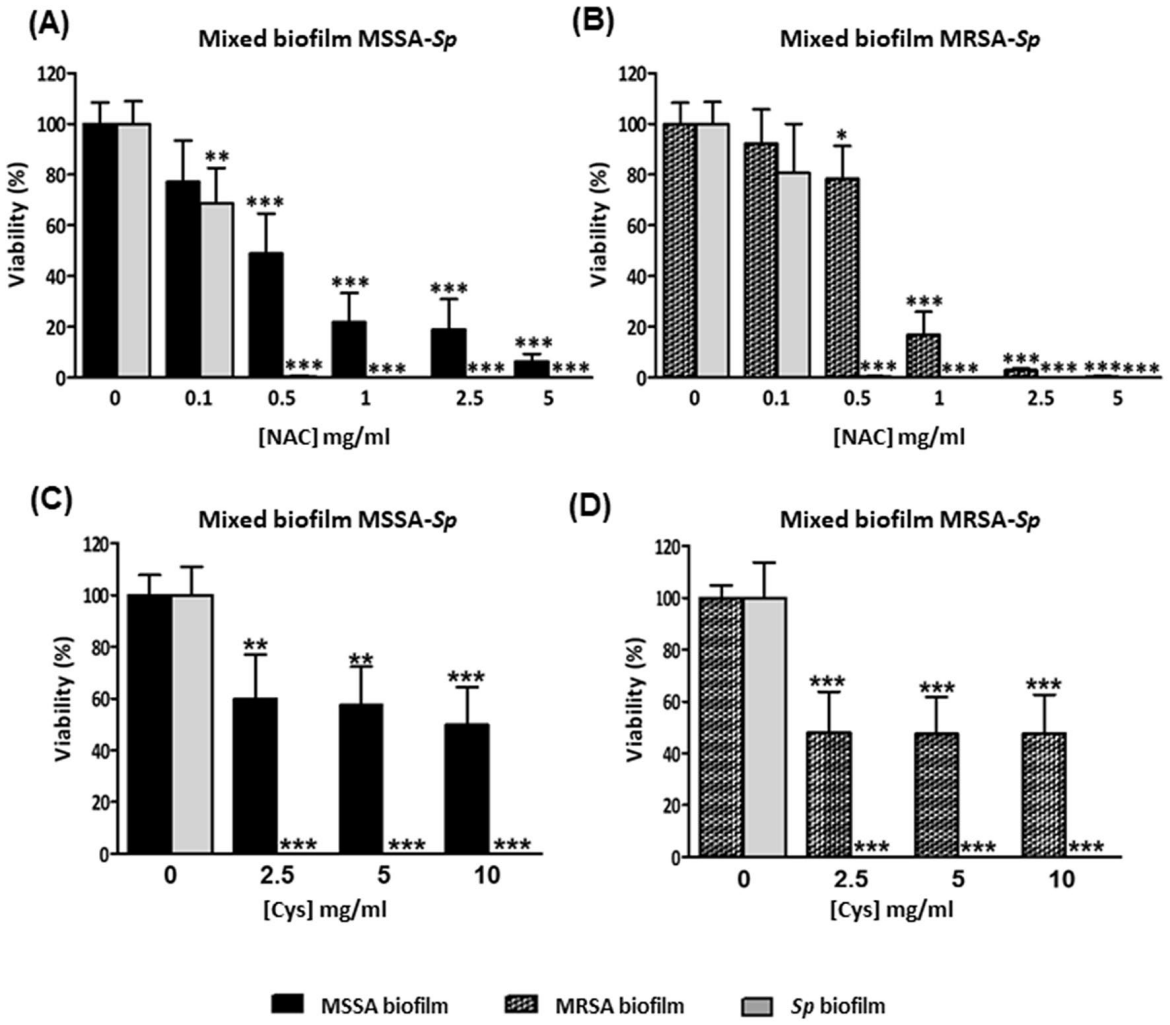
Treatment with antioxidants of MSSA/MRSA-*Sp* mixed biofilms (Disaggregation of mixed biofilms). Mixed biofilms were first incubated for 4 h at 34 °C, then washed with sterile H_2_Od and incubated with different concentrations of NAC (**A**,**B**) or Cys (**C**,**D**) for 1 h at 37 °C. The MIC of NAC for the *S. pneumoniae* strain was 2.5 mg/ml and 5 mg/ml for both MSSA /MRSA strains whereas the MIC of Cys for the *S. pneumoniae* strain and both *S. aureus* strains was 0.156 mg/ml. (**A**,**C**) Viability of MSSA-*Sp* mixed biofilm. (**B**,**D**) Viability of MRSA-*Sp* mixed biofilm. Black bars correspond to MSSA, grey bars correspond to *S. pneumoniae*, and grey patterned bars represent MRSA viable cells within the mixed biofilms. Data represent the average of at least three experiments. Standard deviation bars are shown, and asterisks mark statistically significant results (two-tailed Student’s *t* test: *P < 0.05; **P < 0.01; ***P < 0.001) when comparing the treatment versus the non-treated biofilm. For multiple comparisons, we performed one-way ANOVA followed by a Dunnett’s post hoc test obtaining ***P < 0.001 in all the cases.

Treatment or disaggregation of mixed biofilms (MSSA/MRSA:*Sp*, 1:11) with NAC was effective in reducing both populations (***P < 0.001, one-way ANOVA) (Fig. 5A,B). Mixed biofilms treated with 0.5 mg/ml of NAC showed a clearance of MSSA cells (around 50%), MRSA cells (over 20%), and a marked reduction of *S. pneumoniae* cells (99%) (Fig. 5A,B). At 5 mg/ml, NAC killed nearly 94% of MSSA cells, 99% of MRSA cells and practically eliminated *S. pneumoniae* indicating that NAC could be a promising option for the treatment of *S. aureus*–*S. pneumoniae* mixed biofilms.

Treatment with Cys showed a marked clearance of pneumococcal population within the mixed biofilm with up to 99% reduction of *S. pneumoniae* using a concentration ≥ 2.5 mg/ml in the mixed biofilms with MSSA or MRSA (***P < 0.001, one-way ANOVA) (Fig. 5C,D). However, treatment with Cys was not as effective in decreasing the viability of *S. aureus* within the mixed biofilm (Fig. 5C,D), obtaining a similar level in the reduction of the populations in comparison to the individual biofilm of MSSA or MRSA of serotype 5 described above (***P < 0.001, one-way ANOVA) (Fig. S3A,B). The use of higher concentrations of Cys (levels around 20 mg/ml) cleared the pneumococcal population within the mixed biofilm but the reduction of *S. aureus* was similar to the lower doses (data not shown).

To confirm the effect of both antioxidants, CLSM was performed in MSSA-*Sp* and MRSA-*Sp* biofilms formed in glass-bottom dishes (Fig. S4). Images confirmed the killing effect of 2.5 mg/ml of NAC and Cys in the mixed biofilm system, being greater the effect of NAC in both systems in agreement with viable counts. Figure S4 shows that adding NAC kills the bacterial cells in the biofilms, but does not have a marked effect on the density of the biofilm. Moreover, the lethal effect of 2.5 mg/ml of Cys in the mixed biofilm MSSA-*Sp* was higher than in the mixed biofilm of MRSA-*Sp* (Fig. S4).

### Impact of cefditoren against mixed biofilms of MSSA-*Sp* and MRSA-*Sp*

**Figure 6:**
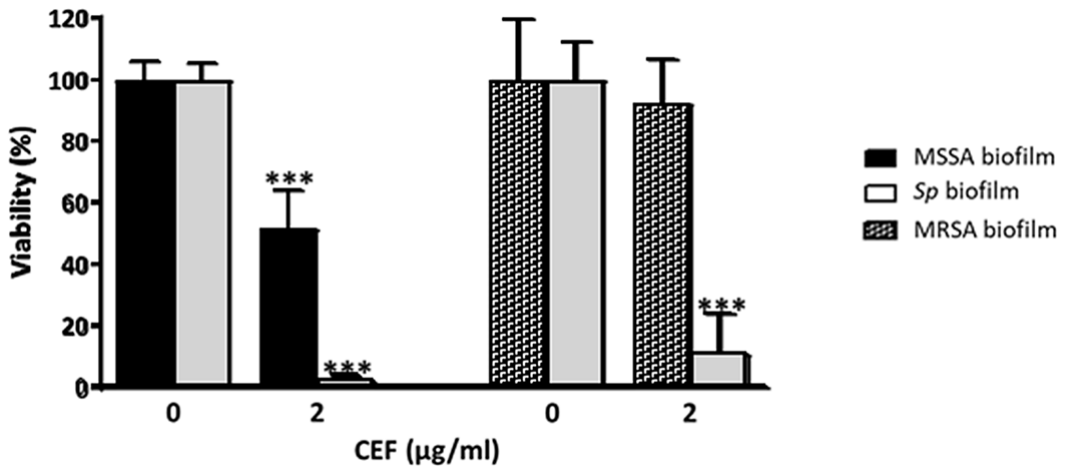
Treatment with CEF of MSSA/MRSA-*Sp* mixed biofilms (Disaggregation of mixed biofilms). Mixed biofilms were first incubated for 4 h at 34 °C, then washed with sterile H_2_Od and incubated with 2 µg/ml of CEF for 1 h at 37 °C. The MIC of CEF for the *S. pneumoniae* strain was 0.12 µg/ml, for the MSSA strain was 1.25 µg/ml and for the MRSA strain was 2.5 µg/ml. Black bars correspond to MSSA, grey bars correspond to *S. pneumoniae*, and grey patterned bars represent MRSA viable cells within the mixed biofilms. Data represent the average of at least three experiments. Standard deviation bars are shown, and asterisks mark statistically significant results (two-tailed Student’s *t* test: ***P < 0.001) when comparing the treatment versus the non-treated biofilm.

The antimicrobial activity of cefditoren (CEF) was used against MSSA-*Sp* and MRSA-*Sp* biofilms. The reason for using this 3rd generation oral cephalosporin is because we have recently demonstrated that it has been the most active β-lactam against *S. pneumoniae* clinical isolates during the last 16 years, demonstrating the lowest MIC_50_ and MIC_90_ throughout the period 2004–2020^55,56^. For biofilm treatment, we used double the MIC_90_ of previous studies of both *S. pneumoniae* clinical isolates and MSSA clinical isolates^55,56^. Our results demonstrated that CEF reduces the pneumococcal population within the mixed biofilm and showed a partial effect against the MSSA strain (Fig. 6). In the case of the mixed MRSA-*Sp* biofilm, this antibiotic only killed the pneumococcal population within the mixed biofilm without effect against the MRSA strain. These results confirm that this antibiotic could be a good choice to ameliorate the impact of pneumococcal resistant strains even when forming complex polymicrobial biofilms, but it is not as effective against the polymicrobial biofilms as the antioxidants NAC and Cys (Figs. 4 and 5).

## Discussion

The existence of polymicrobial biofilms with bacterial species such as *S. aureus* and *S. pneumoniae* is worrisome. Bacteria in biofilms are more recalcitrant to antibiotic treatments and the immune system, therefore facilitating the colonization process. The close contact in the biofilm state contributes to the interchange of virulence factors and resistance genes increasing the relevance of biofilms in clinical practice^26,57–59^. The step from nasopharynx colonization to diseases such as AOM, sinusitis, and bacterial pneumonia is hypothesized by host changes after suffering a viral upper respiratory infection^60–62^. Moreover, epidemiological studies showed a co-colonization in the nasopharynx of humans by *S. aureus* and *S. pneumoniae* despite being traditionally seen as contrasting species^42–44^. This antagonistic relationship has been described previously^39,63^ but our study demonstrates a suitable interaction by both species. The establishment of an in vitro model using 96-well polystyrene plates containing *S. pneumoniae* and *S. aureus* forming a mixed biofilm supports previous works describing the possibility of the interaction between both pathogens^4,40,41^.

In this in vitro model, *S. aureus* has an advantage in the growth as biofilm compared to *S. pneumoniae*, which needs a higher proportion of the inoculum and a higher rate in the planktonic culture to achieve a final concentration within the mixed biofilm similar to the obtained with *S. aureus*. This may happen because of the innate nature of the pathogen that forms biofilms easily in both biotic and non-biotic surfaces^64^. In the case of abiotic surfaces, where electrostatic and hydrophobic interactions play an important role in static biofilm assays, *S. aureus* also uses negatively charged teichoic acids and its major autolysin AltA to attach to polystyrene and glass surfaces^65–67^. In the case of *S. pneumoniae*, the initial attachment to abiotic surfaces has been only reported to be mediated by weak electrostatic and hydrophobic interactions^58,68^. Moreover, the CPS of the pneumococcal strain used, despite expressing the CPS of a good biofilm former serotype^18^, could interfere with the initial attachment and biofilm formation, and therefore, *S. aureus* could take advantage and dominate the attachment and formation within the mixed biofilm.

In this study, we have used two strains with different backgrounds, including MSSA and MRSA clinical isolates, to see if the antibiotic susceptibility profile interferes in the interaction between *S. pneumoniae and S. aureus*. Our results demonstrated that both strains had a similar biofilm pattern individually or mixed with *S. pneumoniae*.

One of the main challenges in the outcome of the infection caused by *S. aureus* biofilms is the loss of efficacy of the antibiotic treatment, especially when these biofilms are formed by MRSA strains^26,69^. It is common that conventional antibiotic therapy does not work in recalcitrant MSSA and MRSA biofilms. This could aggravate the situation, imposing a positive selection pressure for the emergence of resistant strains^6,70^. Treatment with the thiol antioxidant NAC (a precursor of glutathione synthesis), has been used to prevent and disrupt *S. aureus* biofilms, as a single treatment or in combination with other enzymes and antibiotics^49^. Different mechanisms have been reported for the antimicrobial activity of NAC^50,71,72^. Among them, competitive inhibition of cysteine uptake with microbial strains is of great importance. In addition, reaction of the NAC sulfhydryl group with bacterial proteins leading to reduction of disulfide bonds affecting bacterial attachment within the biofilm has been found to reduce bacterial viability. Moreover, modification of the intracellular redox equilibrium also confers antimicrobial activity^50,71^. In addition, NAC could be acting as a weak acid on the biofilm, penetrating the matrix and the cell wall. In this sense, NAC dissociates and acidifies the cytoplasm, denaturing bacterial proteins and causing DNA damage inside the bacteria^72^. Cys is an aminothiol that deprotonates and forms thiol anions (_−_*S*^−^) that are able to disrupt intermolecular and intramolecular disulfide bonds of bacterial proteins^73^. In this case, cleavage of disulfide bonds of bacterial proteins not only denatures key bacterial enzymes that play important roles in their metabolism and survival but also impairs the structural integrity of the extracellular matrix of biofilms^52^. As a consequence of these antimicrobial mechanisms, Cys prevents the formation and disrupts the biofilm of several pathogens including *Pseudomonas aeruginosa*, *Enterococcus faecalis*, *Haemophilus influenzae,* and *S. pneumoniae*^51–53^. In this work, we have demonstrated the antimicrobial effect of different concentrations of Cys in *S. aureus* biofilms, confirming that this compound prevented the biofilm formation of a MSSA and a MRSA strain. Moreover, treatment of *S. aureus* biofilms with doses of Cys over 2.5 mg/ml resulted in a ≈ 50–90% reduction of the viable bacteria of the biofilm, showing a lower effect than NAC for this kind of biofilms^49^. One of the main advantages of this study is the use of relevant clinical isolates of *S. aureus* confirming that both antioxidants are effective against MSSA and MRSA strains. We could observe that methicillin resistance does not change the effect of the mucolytic agent, as it had been previously reported with NAC^49^. To this date, the identification of *S. aureus* strains associated with resistance or even tolerance to NAC/Cys has not been shown.

Prevention of mixed biofilms by these two pathogens and the treatment of polymicrobial biofilms associated with certain diseases such as AOM and sinusitis are strategies to be prioritized. NAC and Cys have been confirmed as good candidates to treat polymicrobial biofilms of *S. pneumoniae* and non-typeable *H. influenzae*^53^, making them ideal to treat biofilms where *S. aureus*, and specifically MRSA, is involved. In this work, we describe the antimicrobial effect of both antioxidants against mixed biofilms of *S. aureus* and *S. pneumoniae*, demonstrating that the pneumococcal population was practically eradicated with only 0.5 mg/ml of NAC and 2.5 mg/ml of Cys. In the case of *S. aureus* in the mixed biofilm, NAC markedly reduced the viability and this effect was independent of the methicillin susceptibility pattern. Moreover, we observed that the treatment with NAC of mixed biofilms does not disperse the bacterial cells within the biofilm. This is an advantage in clinical practice as the not dispersing effect avoids the potential colonization of a new habitat within the host. The antimicrobial activity of Cys against *S. aureus* in the mixed biofilm was similar to the treatment of individual biofilms of the same serotype, preventing mixed biofilms with concentrations around the MIC. Our results showed a weaker effect of Cys compared to NAC, which has been already seen in the treatment of polymicrobial biofilms of non-typeable *H. influenzae* and *S. pneumoniae*^53^. We also used a 3rd generation oral cephalosporin to test a classical antibiotic against polymicrobial biofilms, observing only an effect in the *S. pneumoniae* population using a concentration 16 times over MIC, a mild effect against MSSA population, and non against the MRSA population within the mixed. This confirms that the use of alternative therapies against polymicrobial biofilms such as antioxidants is necessary.

The search for new treatments against bacteria multiplying as biofilms is an urgent matter, and of great importance in the clinical practice which is a growing research field. The development of new drugs is an expensive and slow process, but drug repurposing has been adopted successfully and is a current priority by the National Institutes of Health^54^. Our results contribute to the knowledge supporting the use of NAC and Cys as promising repurposed drugs candidates for the prevention and treatment of individual *S. aureus* biofilms and against mixed biofilms by *S. pneumoniae and S. aureus* including MRSA strains.

## Methods

### Bacterial strains and culture conditions

In mixed biofilms, we tested the following strains: *S. pneumoniae* YNM4 (serotype 19A)^8^, MSSA 60031/19 strain (serotype 5), and MRSA 60061/19 strain (serotype 5). For individual biofilms of *S. aureus* testing Cys, we also used MSSA 60335/19 strain (serotype 8) and MRSA 60221/19 (serotype 8). The pneumococcal strain was cultured in 5% Mueller–Hinton blood agar plates and incubated at 37 °C under 5% CO_2_. *S. aureus* strains were cultured in Tryptic Soy Agar plates and incubated at 37 °C. In mixed infections, we used blood agar plates with 5 µg/ml of gentamicin and plates containing Salt Mannitol Agar to select *S. pneumoniae* and *S. aureus* respectively.

### Mixed biofilm formation assay

Biofilm formation was characterized using a crystal violet (CV) assay as previously described^14^. Briefly, cells were grown in a C + Y medium to an *A*_550_ of ≈ 0.5–0.6 (≈ 4 × 10^8^ CFU/ml) and diluted 100-fold in C + Y medium. *S. pneumoniae* and *S. aureus* suspensions were used individually or combined in different proportions, and aliquots of 200 µl containing from 4 × 10^5^ to 4 × 10^6^ CFU/ml were added into 96-well polystyrene plates (Costar 3595, Corning). The biofilms were incubated from 2 to 6 h at 34 °C and the *A*_595_ of the total growth was measured using the BioTek Epoch2 (BioTek Instruments). This temperature was chosen because it mimics the environment found in the upper respiratory tract when temperatures are a bit cooler due to higher ventilation in this location^74^. CV (0.2%) was used to stain the biofilm followed by three washes with distilled water to eliminate non-adherent bacteria and solubilization with 95% ethanol. Absorbance (*A*_595_) was quantified in the mentioned reader. The number of viable cells (biofilm and planktonic cells) was determined in the different cultures in order to isolate *S. pneumoniae* from *S. aureus* using specific selective plates described above. Briefly, after incubation, the planktonic culture was separated, and the biofilm was rinsed twice with phosphate buffer saline (PBS), then gently disaggregated using a pipette and tenfold dilutions were prepared in PBS. Viable cells were quantified and expressed as CFU/ml.

### Susceptibility testing and antibiofilm therapy

The susceptibility of *S. aureus* and *S. pneumoniae* strains growing as planktonic cultures to the compounds NAC (Sigma-Aldrich) and Cys (Sigma-Aldrich) were determined using the broth microdilution method following CLSI guidelines^75^. The MIC of NAC for the *S. pneumoniae* strain was 2.5 mg/ml and 5 mg/ml for both MSSA /MRSA strains whereas the MIC of Cys for the *S. pneumoniae* strain and for all *S. aureus* strains was 0.156 mg/ml. The MIC of CEF for the *S. pneumoniae* strain was 0.12 µg/ml, for the MSSA strain was 1.25 µg/ml and for the MRSA strain was 2.5 µg/ml.

For the antibiofilm therapy, bacterial cells were grown in a C + Y medium to an *A*_550_ of ≈ 0.5–0.6. Cells were centrifuged and resuspended in an equal volume of C + Y, using a 100-fold dilution in C + Y medium for further work. The suspensions of *S. aureus* were used individually or mixed with *S. pneumoniae* in the proportion 1:1 (for inhibition assays, prevention) or 1:11 (for disaggregation assays, treatment) of MSSA:*Sp* and MRSA:*Sp*. 200 µl of the different bacterial suspensions were added to each well using 96 wells of polystyrene microtiter plates.

For inhibition assays (prevention), different concentrations of NAC and Cys were added to the initial biofilm with the inoculum followed by 5 h incubation at 34 °C. Absorbance (*A*_595_) of the total growth was determined using the BioTek Epoch2. Biofilm staining with CV and quantification was performed as described above.

For disaggregation assays (treatment), after 4 h incubation at 34 °C, the planktonic cells were aspirated and the biofilm was rinsed with PBS. Different concentrations of NAC and Cys were added and biofilms were incubated for 1 h at 37 °C, rinsed twice with PBS, disaggregated, and tenfold dilutions were prepared in PBS. Viable cells were quantified and expressed as CFU/ml.

### Confocal laser scanning microscopy (CLSM) of biofilms

To evaluate the effect of the antioxidants NAC and Cys in the mixed biofilms, we used CLSM to visualize the cells as previously described^53^. Briefly, biofilms were grown on glass-bottomed dishes (WillCo-dish; WillCo Wells B.V., The Netherlands) for 4 h at 34 °C. The supernatant was removed and the biofilm was rinsed with PBS, and treated with 2.5 mg/ml of NAC or Cys for 1 h at 37 °C. Non-adherent bacteria were removed from biofilms by washing with sterile water, and bacterial viability within the biofilm was assessed using the LIVE/DEAD *Bac*Light kit (Invitrogen). CLSM observations were made with a Leica spectral SP5 confocal microscope and analyzed with the LAS AF software. Images represent the x–y from XYZ-stacks at 0.5-µm intervals and x–z projections from XZY stacks at 5-µm intervals planes.

### Statistical analysis

Data were obtained from different independent experiments, containing at least three replicates in each experiment. A two-tailed Student’s *t* test was used for two groups’ comparisons, whereas for multiple comparisons we choose a one-way ANOVA test and a Dunnett’s post hoc test. GraphPad InStat version 8.0 was used for every analysis. We consider *P* < 0.05 (*) as significant whereas *P* < 0.01 (**) and *P* < 0.001 (***) were considered as highly significant.

## Supplementary Information

Supplementary Figures.
