# Supplementary material for: Clearance of mixed biofilms of Streptococcus pneumoniae and methicillin-susceptible/resistant Staphylococcus aureus by antioxidants N-acetyl-l-cysteine and cysteamine
Source: Sci Rep. 2022 Apr 23;12:6668. doi: 10.1038/s41598-022-10609-x (PMC9035182; doi:10.1038/s41598-022-10609-x)
Supplement: Supplementary file 1 — Supplementary Figures. [file 41598_2022_10609_MOESM1_ESM.docx]

**Supplementary figures**

**Fig. S1.** Time course of *S. pneumoniae*, MSSA, and MRSA monospecific biofilm formation. (A) *S. pneumoniae* YNM4 biofilm. In the top panel is represented *S. pneumoniae* (green) viable cells within the biofilm. In the middle panel is represented *S. pneumoniae* (light green) viable cells in the planktonic culture. In the bottom panel is represented the total biomass (black bars) and biofilm biomass (grey bars) determined by CV staining. (B) *S. aureus* 60031/19 MSSA biofilm. In the top panel is represented MSSA (red) viable cells within the biofilm. In the middle panel is represented MSSA (light red) viable cells in the planktonic culture. In the bottom panel is represented the total biomass (black bars) and biofilm biomass (grey bars) determined by CV staining. (C) *S. aureus* 60061/19 MRSA biofilm. In the top panel is represented MRSA (patterned red) viable cells within the biofilm. In the middle panel is represented MRSA (light patterned red) viable cells in the planktonic culture. In the bottom panel is represented the total biomass (black bars) and biofilm biomass (grey bars) determined by CV staining. The data represent the average of six experiments. Standard deviation bars are shown.

**Fig. S2.** Prevention with Cys of monospecific *S. aureus* biofilms (inhibition assays). The different concentrations of Cys were added with the inoculum and then incubated for 5 h at 34°C. After the incubation, CV staining was performed. Dark grey bars represent total biomass (adherent plus non-adherent cells) and white bars represent biofilm biomass (biofilm of adherent cells). MIC of Cys for all *S. aureus* strains was 0.156 mg/ml. (A, B) Inhibition of MSSA/MRSA strains of serotype 5. (C, D). Inhibition of MSSA/MRSA strains of serotype 8. Data represent the average of at least three experiments. Standard deviation bars are shown and asterisks mark results that are statistically significant (two-tailed Student’s t-test: ******P*<0.05; ********P*<0.001).


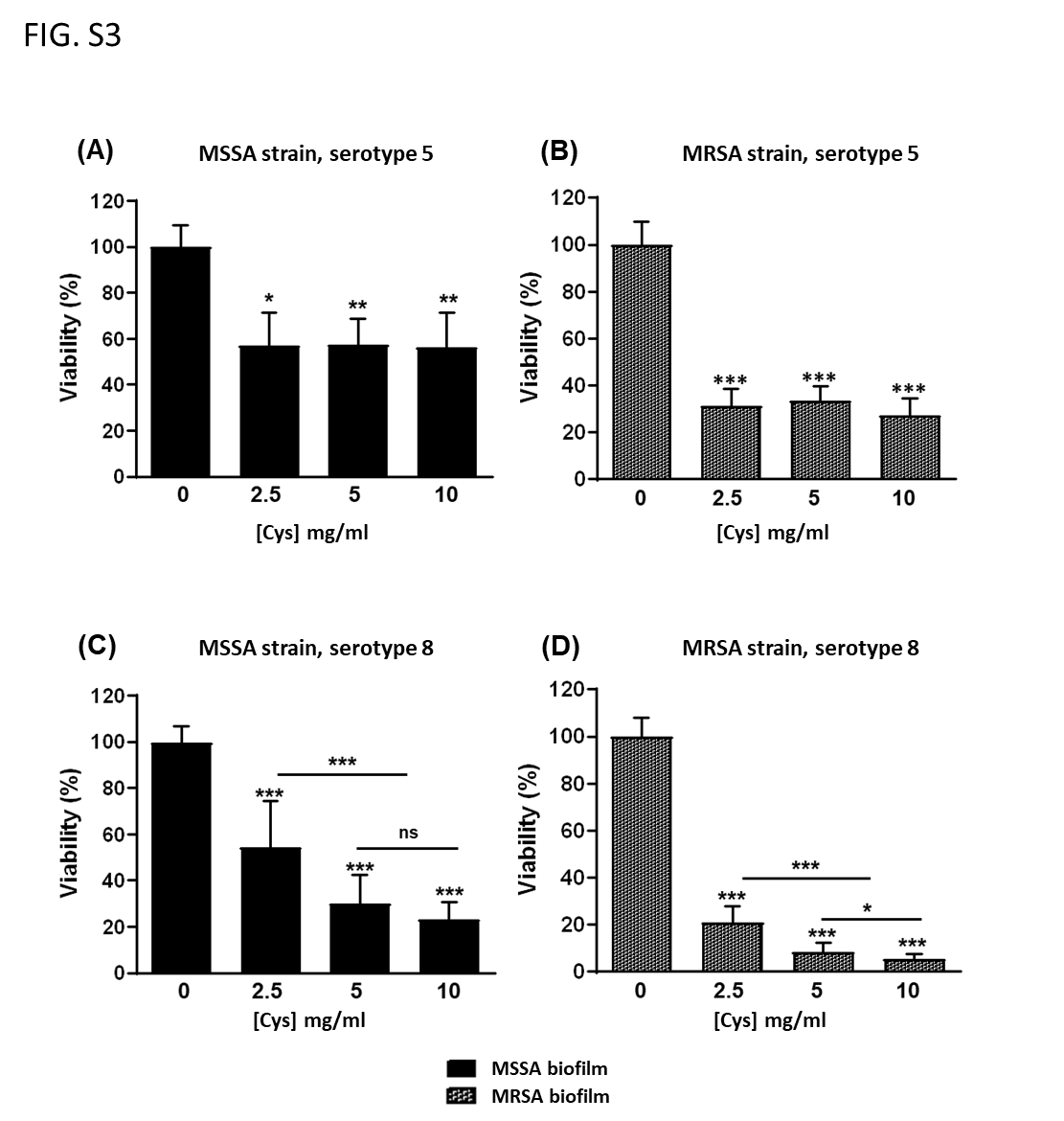


**Fig. S3.** Treatment with Cys of monospecific *S. aureus* biofilms (disaggregation assays). After 4 h of incubation at 34°C, *S. aureus* biofilms were washed with sterile H_2_Od and incubated with different concentrations of Cys for 1 h at 37°C. The viability was determined by plate counting. Black bars correspond to MSSA biofilm viability and grey patterned bars represent MRSA biofilm viability. MIC of Cys for all *S. aureus* strains was 0.156 mg/ml. (A, B) Treatment of MSSA/MRSA strains of serotype 5. (C, D). Treatment of MSSA/MRSA strains of serotype 8. Data represent the average of at least three experiments. Standard deviation bars are shown and asterisks mark results that are statistically significant (two-tailed Student’s t-test: ns, no significant; ******P*<0.05; *******P*<0.01; ********P*<0.001). For multiple comparisons we performed one-way ANOVA followed by a Dunnett’s post hoc test obtaining ********P*<0.001 in all the cases.

**Fig. S4.** CLSM of a MSSA-*Sp* and a MRSA-*Sp* mixed biofilm after exposure to NAC and Cys. A MSSA-*Sp* and a MRSA-*Sp* mixed biofilm were untreated or treated with 2.5 mg/ml Cys or NAC for 1 h at 37°C. The cells in the biofilms were then stained with the *Bac*Light LIVE/DEAD kit to reveal viable (green fluorescence) and non-viable (red fluorescence) bacteria. Projections were obtained in the x-y (individual scans at 0.5 µm intervals) and the x-z (individual scans at 5 µm intervals). Scale bars indicate 25 µm.
